# Experimental study of the Al–Cu–Zn ternary phase diagram

**DOI:** 10.1007/s10853-020-04686-4

**Published:** 2020-04-30

**Authors:** Ondrej Zobac, Ales Kroupa, Klaus W. Richter

**Affiliations:** 1 Department of Inorganic Chemistry - Functional Materials, University of Vienna, Währinger Straße 42, 1090 Vienna, Austria; 2 Institute of Physics of Materials, The Czech Academy of Sciences, Zizkova 22, 616 00 Brno, Czech Republic

## Abstract

The phase diagram of the Al–Cu–Zn ternary system was re-investigated experimentally. The current study was designed to contribute to a better description of those parts of the phase diagram, which are disputed in the current scientific literature. Mutual relations in the family of ternary intermetallic phases *τ* with cubic, rhombohedral and modulated structure at temperatures 400 °C and 550 °C were described. The phase relation between the *γ* and *γ*′ phases was studied at different temperatures. A two-phase field between *γ* and *γ*′ was observed below 400 °C, while the transition appears to be second order at higher temperatures. A vertical section between *γ* and *γ*′ phases in Cu–Zn and Al–Cu and four isothermal sections at 400 °C, 550 °C, 700 °C and 820 °C, respectively, were constructed.

## Introduction and literature review

### Introduction

The Al–Cu–Zn ternary system has been investigated intensively in the past because of its technical importance for developing of light-weight alloys. However, several areas of the phase diagram are still not well understood and discussed intensively. Our work is focused on several doubtful regions of the phase diagram, which have not yet been satisfactorily resolved in the scientific literature, and on the divergent results from the experimental and theoretical publications [1, 2]. Major topics were the extensions of the *γ* and *γ*′ solid solution phases (with primitive and base-centered brass structures, respectively) and their mutual relation in the whole concentration and temperature range. We also studied the crystal structure and homogeneity ranges of the reported ternary phases *τ* and *τ*′ and constructed the isothermal sections of the phase diagram Al–Cu–Zn based on our new experimental results at 400 °C, 550 °C, 700 °C and 820 °C as well as the vertical section describing the mutual relation between *γ* and *γ*′ phases.

### Literature review

Detailed literature reviews of all binary subsystems are described in recent literature. The experimental description of the Al–Cu phase diagram is published in our previous work [3]. Liang et al. [4] published a theoretical modeling of the Al–Cu phase diagram based on CALPHAD approach.

The Al–Zn binary phase diagram was assessed by Murray [5] and reported by Massalski [6]. Dinsdale et al. [7] have done the most recent theoretical modeling of the Al–Zn phase diagram, using the third generation of unary data. Theoretical modeling using the commonly used SGTE unary database [8] was published by Mathon et al. [9].

**Figure 1:**
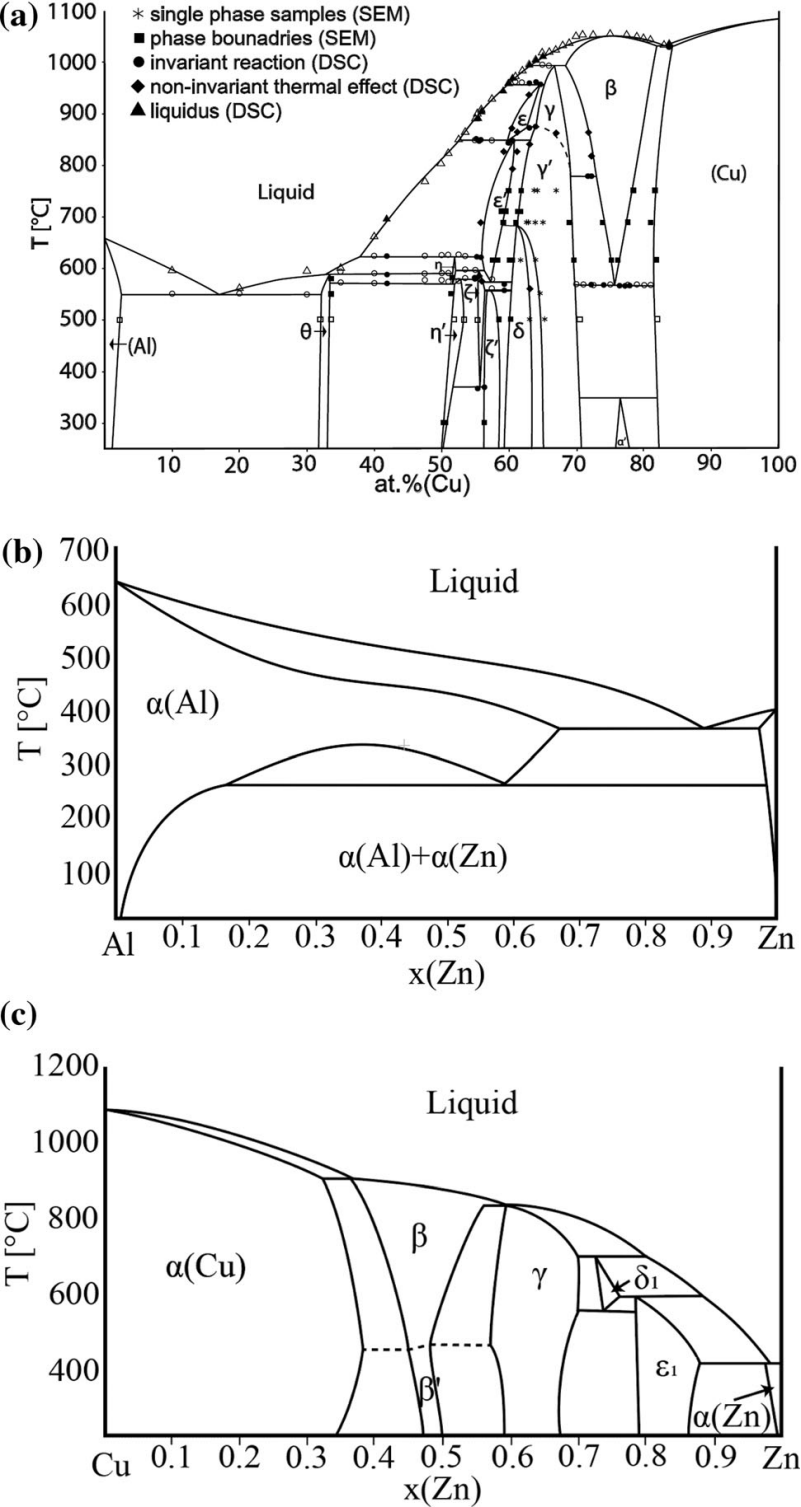
Binary phase diagram of subsystem. **a** Al–Cu [3], **b** Al–Zn [6], **c** Cu–Zn [6]

The assessed Cu–Zn binary phase diagram was published by Miodownik [10]. Liang et al. [11] published the most recent theoretically modeled binary phase diagram Cu–Zn. Figure 1a–c show redrawn binary experimental phase diagrams of the relevant subsystems.

Ghosh et al. [1] published a review of the experimental studies on the Al–Cu–Zn phase diagram available up to the year 2002. The experimental ternary phase diagram Al–Cu–Zn provided in [1] can be generally accepted except for the disagreement in the proposed phase equilibria between the *γ* and *γ*′ phases in comparison with the detailed analysis of existing experimental studies presented by Liang and Schmid-Fetzer [2]. These authors also published the most recent theoretical assessment based on this analysis in the same paper [2]. The main difference exists for mutual relation between the two closely related *γ* (Al_4_Cu_9_) and *γ*′(Cu_5_Zn_8_) phases and ternary *τ* and *τ*′ phases which will be discussed later. Most of the original references from the years 1905–2002 evaluated in Ghosh et al. [1] are not duplicated here.

Liang and Chang [12] published an overall thermodynamic description based on the CALPHAD approach. Two isothermal sections at 500 °C and 700 °C and a liquidus projection were published in this paper. Only one ternary intermetallic compound *τ* (approx. formula Al_3_Cu_5_Zn_2_) was theoretically modeled in this system by Liang and Chang [12]. Despite the fact that the experimentally established homogeneity range of the *τ* phase is highly temperature dependent, Liang and Schmid-Fetzer [2] modeled the *τ* phase as a linear phase with constant composition x(Zn) = 0.10. It was found earlier that the family of *τ*-phases appears in two modifications, cubic B2-type *τ* phase and a structurally related rhombohedral *τ*′ phase [13]. The *τ*′ phase was not included into Liang’s [2, 12] thermodynamic description because the composition range and thermal stability had not yet been reasonably determined.

The continuous solid solutions were modeled between some binary intermetallic phases in this system. The β (BCC_A2) phase exists in both Al–Cu and Cu–Zn systems and the continuous solid solution was modeled in [12]. The situation is more complicated in the case of the family of *γ*-phases. The *γ* phase region forms continuous solid solution in all experimentally assessed isothermal sections from 350 °C to 700 °C [1], from the Al–Cu side, where the low temperature *γ*′ (Al_4_Cu_9_) with the Pearson symbol *cP*52 exists in the binary Al–Cu system, to the Cu–Zn side where the *γ* (Cu_5_Zn_8_) phase with the Pearson symbol *cI*52 is stable.

The first results showing continuous *γ* phase region between Al–Cu and Cu–Zn binaries were presented by Bauer and Hansen [14]. They constructed several isothermal sections of Al–Cu–Zn ternary system in the Cu-rich corner in temperature range 410–800 °C. They proposed the continuous solid solution of the *γ* phase based on their experimental results. Koster and Moeller [13] constructed an isothermal section at 350 °C and confirmed the continuous solubility of the *γ* phase. Ashirimbetov et al. [15] reported two isothermal sections at 20 °C and 350 °C. The results showed the solid solubility of Al in *γ* at 20 °C and 350 °C are about 3.5 and 7.0 wt % (2.2 and 5.5 at.%), respectively, and solubility of Zn in *γ*′ at the same temperatures about 30 wt %. They detected a wide two-phase region between *γ* and *γ*′ at room temperature and 350 °C. They predicted that these two different *γ* phases might be miscible at higher temperatures where the high temperature modification of the *γ* phase exists in the Al–Cu system with the same crystal structure as in the Cu–Zn system. Also Liang and Schmid-Fetzer [2] did not accept the continuous solid solution between these crystallographically slightly different types of *γ* phases in their theoretical work. They described this decision in detail in the text of their paper. Nevertheless, they did not run any experimental study to confirm their conclusions.

With respect to previously mentioned result discrepancies, the aim of our study of the Al–Cu–Zn ternary system was to experimentally solve the question of solubilities between the *γ*′ and *γ* phases and a detailed crystallography and compositional and temperature stability of *τ* and *τ*′ phases.

The current available information for all solid phases of the system is summarized in Table 1. The abbreviated phase names in the form of Greek letters used in the text and phase diagrams are provided in the first column. Other common phase names used in the literature [1–3] are shown in column 2. The Pearson symbol and structure type (columns 3 and 4) are taken from the Materials Science International Team (MSIT) report [16]. Temperature stability of binary phases is taken from relevant phase diagrams [3, 6]. Temperature stability of ternary phase *τ* (in our text is equal to *τ*_c_) from Ghosh et al. [1] and ternary phases *τ*_i_ and *τ*_r_ (which corresponds to *τ*′) is based on our new results.

**Table 1:** Stable phases in Al–Cu-Zn ternary phase diagram and binary subsystems

Phase name [This work]	Common names	Pearson symbol	Structure prototype	T. range (°C)
*α* (Al)	FCC_A1, Al	*cF*4	Al	≤ 660.5
*α* (Cu)	FCC_A1, Cu	*cF*4	Cu	≤ 1083
*α* (Zn)	HCP_Zn, Zn	*hP2*	Mg	
*θ*	*θ*, Al_2_Cu	*tI*12	Al_2_Cu	≤ 590.5
*η*	*η*_1__AlCu, Eta HT	*oP*16/*oC*16	n.a.	573.9-624.5
*η*′	*η*_2__AlCu, Eta LT	*mC*20	AlCu	≤ 574.5
ζ	ζ_2_, Al_3_Cu_4-δ_	*Imm*2	Al_3_Cu_4−δ_	373–597
*ζ*′	ζ_1_, Al_3_Cu_4_	*Fmm*2	Al_3_Cu_4_	min. 300–560.5
*ε*	*ε*_1__AlCu, epsilon HT	cubic?	n.a.	959–846
*ε*′	*ε*_2__AlCu, epsilon LT	*hP*4	NiAs	846–568.5
δ	δ, Al_5_Cu_8_	*hR*52	Al_4_Cu_9_ (r)	≤ 680
*γ*	*γ*_0_, *γ*_CuZn, *γ*_brass	*cI*52	Cu_5_Zn_8_	991–779.6
*γ*′	*γ*_1_, *γ*___AlCu, *γ*_D8_3_	*cP*52	Al_4_Cu_9_	≤ 873.5
β	BCC_A2 (AlCu)	*cI*2	W	1052–566.7
*α*’	*α*_2__AlCu, alpha_LT	n. a.	super structure based on TiAl_3_	≤ 360
β’	BCC_B2, CuZn	*cP*2	CsCl	≤ 468
δ_1_	CuZn_3_	*hP*3	CuZn_3_	700–560
*ε*_1_	CuZn_4_	*hP*2	Mg	≤598
*τ*_c_	*τ*, Cu_5_Zn_2_Al_3_	*cP*2	CsCl	≤ 740
*τ*_i_			Incommensurate *τ* phase	≤ 550*
*τ*_r_	*τ*′	*hR*9	superstructure of CsCl	< 440*

*Based on constructed isothermal sections in this work

## Experimental

The overall compositions of experimental samples were selected in order to address the unsolved questions in the experimental phase diagrams as mentioned above. Furthermore, it was our aim to determine complete isothermal phase equilibria in four isothermal sections. The prepared samples were analyzed and characterized by different static and dynamic analytical methods (SEM–EDX, DTA, XRD).

### Sample preparation

Samples were prepared from pure elements of 5 N purity. Any oxide presented in the copper was reduced by flowing of the H_2_ gas at 300 °C for 3 h. The alloys were re-melted at 950 °C several times in evacuated quartz glass ampoules in order to improve the homogenization of the material. Long-term annealing of the samples was performed at selected temperatures on material sealed in evacuated quartz glass ampoules. To prevent reaction of liquid Al with the Si from material of quartz glass, the samples with high content of Al-rich liquid were placed inside the corundum crucibles. These crucibles with samples were sealed in evacuated quartz glass ampoules. A conventional muffle resistance furnace was used for the heat treatment. Samples were long-term annealed to achieve state close to the thermodynamic equilibrium. Annealing time was selected with respect to annealing temperature. For the annealing temperature closer to the melting temperature, shorter annealing time is sufficient. Annealing was terminated by quenching of the samples into cold water from their annealing temperatures, and sample was prepared for further investigations. Annealing times and temperatures are given in Table 2 together with experimental results.

**Table 2:** Chemical composition of the long-term annealed representative sample

T [°C]_No.	Annealing time [h]	Overall composition (at. %)			Coexist. phases	Phase 1 (at. %)			Phase 2 (at. %)			Phase 3 (at. %)		
		Al	Cu	Zn		Al	Cu	Zn	Al	Cu	Zn	Al	Cu	Zn
300_1	700	14.3	48.8	36.9	*γ* + *γ*′	10.5	44.2	45.3	21.0	53.5	25.5	–	–	–
325_1	700	15.1	48.6	36.3	*γ* + *γ*′	11.0	45.1	43.9	20.1	53.2	26.7	–	–	–
350_1	700	14.9	48.2	36.9	*γ* + *γ*′	11.4	47.0	41.6	18.5	52.4	29.1	–	–	–
375_1	700	16.9	50.8	32.3	*γ* + *γ*′	12.2	46.7	41.1	17.8	52.4	29.8	–	–	–
400_1	648	21.0	39.0	40.0	*τ*_*c*_+* ε*_*1*_	29.9	46.7	23.4	17.9	36.4	45.7	–	–	–
400_2	648	42.4	52.3	5.3	*ζ *+* η*′	45.3	51.7	3.0	41.9	52.0	6.1	–	–	–
400_3	648	14.3	61.6	24.1	*γ*′ + β’	22.2	61.7	16.1	10.9	61.8	27.3	–	–	–
400_4	648	53.6	42.5	3.9	*η*′+* τ*_*r*_+* θ*	47.8	50.2	2.0	55.3	40.0	4.7	65.4	34.0	0.6
400_5	648	47.6	47.7	4.8	*η*′+* τ*_*r*_	46.59	49.61	3.80	53.1	41.0	5.9	–	–	–
400_6	648	26.1	34.8	39.1	*τ*_i_ + *ε*_1_	42.3	42.6	15.1	13.3	30.3	56.4	–	–	–
400_7	648	18.6	42.9	38.5	*τ*_c_ + *γ* + *ε*_1_	25.6	49.1	25.3	15.7	44.4	39.9	15.4	36.8	47.8
400_8	648	32.8	47.8	19.4	*τ*_*c*_	32.8	47.8	19.4	–	–	–	–	–	–
400_9	648	37.4	46.0	16.6	*τ*_*i*_	37.4	46.0	16.6	–	–	–	–	–	–
400_10	648	42.4	44.1	13.5	*τ*_*i*_	42.4	44.1	13.5	–	–	–	–	–	–
400_11	625	48.4	41.7	9.9	*τ*_*r*_	48.4	41.7	9.9	–	–	–	–	–	–
400_12	625	29.1	57.5	13.4	*δ *+* τ*_*c*_	29.4	59.3	11.3	28.4	50.9	20.7	–	–	–
400_13	625	38.6	55.8	5.6	*δ *+* ζ*′	35.8	59.8	4.4	41.0	52.5	6.5	–	–	–
400_14	625	15.8	49.7	34.5	*γ *+* γ′*	15.8	49.2	35.0	17.4	51.0	31.6	–	–	–
400_15	625	13.8	48.2	38.0	*γ*	13.8	48.2	38.0	–	–	–	–	–	–
400_16	625	11.8	46.3	41.9	*γ*	11.8	46.3	41.9	–	–	–	–	–	–
400_17	612	16.4	51.6	32.0	*γ′*	16.4	51.6	32.0	–	–	–	–	–	–
400_18	612	42.9	48.3	8.8	*η*′+* τ*_*r*_	42.9	49.6	7.5	44.7	43.8	11.5	–	–	–
400_19	612	38.2	50.7	11.1	*ζ*′+* τ*_*i*_+* δ*	40.4	50.3	9.4	34.3	48.4	17.3	–	–	–
400_20	612	31.9	58.0	10.1	*δ *+* τ*_*c*_	32.0	59.5	8.5	30.3	51.0	18.7	–	–	–
400_21	612	28.1	40.8	31.1	*τ*_i_ + *ε*_1_	35.6	46.1	18.3	13.4	32.0	54.6	–	–	–
400_22	612	9.8	36.6	53.6	*γ* + *ε*_1_	9.5	40.2	50.4	10.5	32.4	57.0	–	–	–
400_23	612	5.7	55.3	39.0	β	5.7	55.3	39.0	–	–	–	–	–	–
400_24	612	37.5	14.8	47.7	*τ*_r_ + L	51.1	40.2	8.7	35.0	8.6	56.4	–	–	–
400_25	660	29.4	21.8	48.8	*τ*_r_ + L+*ε*_1_	50.3	40.3	9.4	31.0	8.0	61.0	8.8	23.6	67.6
400_26	660	56.7	4.6	38.7	*α* (Al)	56.7	4.6	38.7	–	–	–	–	–	–
400_27	660	38.0	34.0	28.0	*τ*_r_ + *ε*_1_ + L	50.3	39.8	9.9	10.6	25.8	63.6	–	–	–
400_28	660	23.2	15.3	61.5	*ε*_1_ +L	9.96	23.12	66.92	32.3	8.1	59.6	–	–	–
400_29	660	6.1	34.3	59.6	*γ* + *ε*_1_	6.6	35.7	57.7	4.7	26.3	69.0	–	–	–
400_30	660	21.7	67.9	10.4	*γ*′ + *α* (Cu)	25.7	64.7	9.6	12.3	74.2	13.5	–	–	–
400_31	660	18.0	59.8	22.2	*γ*′ + β	20.3	59.6	20.1	10.7	59.1	30.2	–	–	–
400_32	660	14.4	52.1	33.5	*γ′*	14.4	52.1	33.5				–	–	–
400_33	660	17.0	50.2	32.8	*γ *+* γ′*	15.4	48.7	35.9	18.2	50.7	31.1	–	–	–
400_34	715	50.9	40.3	8.8	*τ*_*r*_	50.9	40.3	8.8				–	–	–
400_35	715	74.8	18.8	6.4	*θ* + *α* (Al)	65.7	32.5	1.8	85.2	2.1	12.7	–	–	–
400_36	715	69.2	19.2	11.6	*θ* + *α* (Al)	65.7	31.7	2.6	78.8	3.1	18.1	–	–	–
400_37	715	26.6	6.3	67.1	L	26.5	6.3	67.2				–	–	–
400_38	715	18.6	53.7	27.7	*γ*′	18.6	53.7	27.7				–	–	–
400_39	715	63.8	18.0	18.2	*θ* + *α* (Al)	65.8	31.5	2.7	60.3	4.1	35.4	–	–	–
400_40	715	28.8	37.8	33.4	*τ*_i_ + *ε*_1_	40.2	43.5	16.3	13.3	31.6	55.1	–	–	–
400_41	715	59.4	20.9	19.7	*θ* + *τ*_r_ + *α* (Al)	65.8	31.9	2.3	53.5	38.7	7.8	54.0	4.7	41.3
400_42	680	51.1	25.1	23.8	*τ*_r_ + *α* (Al) + L	52.7	39.0	8.3	50.3	4.7	45.0	30.0	9.8	60.2
400_43	680	48.6	26.7	24.7	*τ*_r_ + *α* (Al) + L	51.7	39.9	8.4	48.7	4.6	46.7	30.3	8.5	61.2
400_44	680	34.1	54.8	11.1	*δ *+* ζ*′+* τ*_*c*_	32.8	60.2	7.0	38.9	51.5	9.6	32.0	50.7	17.3
400_45	680	3.5	8.3	88.2	*ε*_1_ + L	1.7	15.1	83.2	4.6	3.1	92.3	–	–	–
400_46	680	30.4	49.0	20.6	*τ*_*c*_	30.4	49.0	20.6				–	–	–
400_47	680	45.4	43.6	11.0	*τ*_*i*_	45.4	43.6	11.0				–	–	–
400_48	680	39.9	44.6	15.5	*τ*_*i*_	39.8	44.7	15.5				–	–	–
400_49	680	35.0	46.1	18.9	*τ*_*i*_	35.0	46.1	18.9				–	–	–
400_50	680	28.9	49.4	21.7	*τ*_*c*_	28.9	49.4	21.7				–	–	–
450_1	500	15.9	50.9	33.2	*γ*/*γ*′	15.9	50.1	34.0	16.2	50.5	33.3	–	–	–
500_1	500	16.0	49.3	34.7	*γ*/*γ*′	15.3	52.2	32.5				–	–	–
550_1	840	32.5	55.2	12.3	*γ*′ + *τ*_c_	31.9	59.1	9.0	32.8	53.0	14.2	–	–	–
550_2	840	29.5	55.5	15.0	*γ′ *+* τ*_*c*_	29.3	57.5	13.2	30.3	52.1	17.6	–	–	–
550_3	840	21.7	55.4	22.9	*γ′*	21.7	55.4	22.9				–	–	–
550_4	840	10.5	55.8	33.7	β + *γ*′	8.3	58.5	33.2	11.7	54.5	33.8	–	–	–
550_5	840	36.0	56.1	7.9	*δ *+* τ*_*i*_	35.4	58.6	6.0	36.3	53.4	10.3	–	–	–
550_6	840	31.8	41.0	27.2	*τ*_*c*_+ *L*+*ε*_*1*_	33.9	46.7	19.4	43.4	30.9	25.7	26.1	42.5	31.4
550_7	840	5.9	39.8	54.3	*γ*	5.9	39.8	54.3	–	–	–	–	–	–
550_8	840	5.4	52.5	42.1	β + *γ*′	4.5	55.2	40.3	6.6	49.0	44.4	–	–	–
550_9	840	24.3	45.5	30.2	*ε*_*1*+_*γ*	24.3	45.5	30.2	–	–	–	–	–	–
550_10	840	4.5	65.3	30.2	*α* (Cu) + β	4.1	67.0	28.9	6.2	62.3	31.5	–	–	–
550_11	840	22.6	47.4	30.0	*τ*_c_ + *γ* + δ	26.6	50.0	23.4	19.2	48.6	32.2	23.9	45.4	30.7
550_12	420	23.7	69.9	6.4	β + *γ*′	20.4	72.6	7.0	27.6	67.0	5.4	–	–	–
550_13	420	20.2	65.9	13.9	β + *γ*′	25.1	63.5	11.4	16.2	67.6	16.2	–	–	–
550_14	420	10.2	71.3	18.5	*α* (Cu) + β	9.4	72.0	18.6	13.2	67.6	19.2	–	–	–
550_15	420	40.6	48.1	11.3	*τ*_*i*_	40.6	48.1	11.3	–	–	–	–	–	–
550_16	420	35.1	48.4	16.5	*τ*_*c*_	35.1	48.4	16.5	–	–	–	–	–	–
550_17	420	41.3	43.8	14.9	*τ*_i_ + L	40.6	44.4	15.0	58.7	29.5	11.8	–	–	–
550_18	420	27.5	47.1	25.4	*τ*_c_ + *ε*_1_	29.6	49.9	20.5	26.7	46.4	26.9	–	–	–
550_19	420	30.3	34.4	35.3	*ε*_1_ + L	25.3	42.5	32.2	36.9	23.7	39.4	–	–	–
550_20	420	41.6	47.8	10.6	*τ*_*i*_	41.6	47.8	10.6	–	–	–	–	–	–
550_21	560	56.8	40.3	2.9	*τ*_r_ + *θ*	49.9	45.4	4.7	65.2	34.0	0.8	–	–	–
550_22	560	46.7	46.1	7.2	*τ*_*i*_	46.7	46.1	7.2	–	–	–	–	–	–
550_23	560	37.4	51.9	10.7	*τ*_*i*_	37.4	51.9	10.7	–	–	–	–	–	–
550_24	560	12.4	46.3	41.3	*γ/γ′*	12.4	46.3	41.3	–	–	–	–	–	–
550_25	560	9.5	50.1	40.4	*γ*	9.5	50.1	40.4	–	–	–	–	–	–
550_26	560	9.5	47.1	43.4	*γ*	9.4	47.2	43.4	–	–	–	–	–	–
550_27	560	24.7	73.9	1.4	*α* (Cu) + *γ*′	18.3	80.3	1.4	28.9	69.8	1.3	–	–	–
550_28	560	53.3	39.2	7.5	*τ*_i_ + L	47.1	45.5	7.4	62.5	29.6	7.9	–	–	–
550_29	635	25.8	32.3	41.9	*ε*_*1*_+* L*	21.9	39.2	38.9	31.9	19.2	48.9	–	–	–
550_30	635	25.2	49.4	25.4	*τ*_*c*_+* γ′*	28.2	49.8	22.0	22.7	49.1	28.2	–	–	–
550_31	635	6.2	31.5	62.3	*ε*_*1*_+* γ*	6.5	33.0	60.5	3.8	26.5	69.7	–	–	–
550_32	635	39.4	57.9	2.7	*δ *+* ζ*′	37.7	59.7	2.6	39.8	57.6	2.6	–	–	–
550_33	635	16.8	39.6	43.6	*γ *+* δ*_*1*_	16.6	41.2	42.2	17.1	39.2	43.7	–	–	–
550_34	635	42.6	54.5	2.9	ζ	42.6	54.5	2.9				–	–	–
550_35	635	16.4	40.3	43.3	*γ *+* δ*_*1*_	15.2	42.1	42.7	16.1	39.1	44.8	–	–	–
550_36	635	3.8	30.0	66.2	*ε*_*1*_+* γ*	4.2	34.9	60.9	3.9	30.7	65.4	–	–	–
550_37	635	11.6	36.1	52.3	*ε*_*1*_+* γ*	11.3	36.1	52.6	12.1	33.5	54.4	–	–	–
550_38	635	21.3	45.2	33.5	*γ *+* δ *+* ε*_*1*_	19.1	47.4	33.5	22.0	43.4	34.6	–	–	–
700-_1	570	40.26	57.87	1.87	*ε*′	40.26	57.87	1.87				–	–	–
700_2	570	38.4	56.8	4.8	*γ′ *+* ε*′	*	*	*	38.4	56.8	4.8	–	–	–
700_3	570	33.3	59.1	7.6	*γ′ *+* ε*′	33.3	59.1	7.6	*	*	*	–	–	–
700_4	570	32.8	56.8	10.4	*γ*′	32.8	56.8	10.4				–	–	–
700_5	570	30.4	56.9	12.7	*γ *+* τ*_*c*_	29.7	59.5	10.8	30.8	56.3	12.9	–	–	–
700_6	570	27.9	57.5	14.6	*γ *+* τ*_*c*_	27.7	57.7	14.6	*	*	*	–	–	–
700_7	570	24.4	55.7	19.9	*γ*	24.4	55.7	19.9				–	–	–
700_8	570	17.5	54.7	27.8	*γ*	17.5	54.7	27.8	–	–	–	–	–	–
700_9	450	35.5	58.4	6.1	*γ′ *+* ε*′	34.4	59.9	5.7	35.6	58.3	6.1	–	–	–
700_10	450	33.6	56.1	10.3	*γ′ *+* τ*_*c*_	32.0	58.6	9.4	33.6	55.7	10.7	–	–	–
700_11	450	13.4	73.4	13.2	*α* (Cu) + β	12.9	73.6	13.5	16.0	70.5	13.5	–	–	–
700_12	450	12.5	57.1	30.4	β + *γ*	12.5	57.6	29.9	14.5	54.8	30.7	–	–	–
700_13	450	40.7	44.8	14.5	*τ*_*c*_+* L*	34.3	51.5	14.2	45.3	39.7	15.0	–	–	–
700_14	480	33.7	43.4	22.9	*τ*_*c*_+* L*	28.9	49.0	22.1	40.9	36.5	22.6	–	–	–
700_15	480	24.8	43.0	32.2	*ε*_*1*_+* L*	24.6	42.8	32.6	33.3	28.7	38.0	–	–	–
700_16	480	16.3	35.2	48.5	*ε*_*1*_+* L*	16.2	39.4	44.4	16.6	28.0	55.4	–	–	–
700_17	480	12.5	32.6	54.9	*ε*_*1*_+* L*	13.0	35.6	51.4	12.6	22.3	65.1	–	–	–
700_18	480	8.1	28.2	63.7	*ε*_*1*_+* L*	7.8	32.3	59.9	9.2	23.4	67.4	–	–	–
820_1	330	33.8	59.3	6.9	*γ′ *+* ε*	32.9	60.8	6.3	34.2	59.0	6.8	–	–	–
820_2	330	19.6	61.1	19.3	*γ*	19.6	61.1	19.3	–	–	–	–	–	–
820_3	330	36.5	60.3	3.2	*γ′ *+* ε*′	35.2	61.7	3.1	37.2	59.6	3.2	–	–	–
820_4	330	28.6	57.9	13.5	*γ *+* L*	27.7	59.1	13.2	29.4	54.9	15.7	–	–	–
820_5	330	24.8	52.6	22.6	*γ *+* L*	24.3	53.4	22.3	25.3	51.6	23.1	–	–	–
820_6	330	17.8	70.1	12.1	β	17.8	70.1	12.1	–	–	–	–	–	–
820_7	330	30.0	61.6	8.4	*γ*	30.0	61.6	8.4	–	–	–	–	–	–
820_8	330	24.8	71.0	4.2	β + *γ*	25.0	70.5	4.5	28.2	67.9	3.9	–	–	–
820_9	330	17.9	64.9	17.2	β	17.9	64.9	17.2	–	–	–	–	–	–
820_10	330	15.2	76.9	7.9	*α* (Cu) +β	14.0	77.9	8.1	17.2	74.5	8.3	–	–	–
820_11	330	30.8	57.0	12.2	*γ *+* L*	30.8	57.6	11.6	32.2	54.1	13.7	–	–	–
820_12	330	9.1	68.6	22.3	*α* (Cu) +β	7.7	70.6	21.7	9.4	66.4	24.2	–	–	–
820_13	330	4.7	53.4	41.9	β	4.7	53.4	41.9	–	–	–	–	–	–

Coexisting phases found by XRD are in italic font. Coexisting phases established from their compositions are in standard font. * composition has not been measured

### Experimental phase diagram investigation

A combination of dynamic and static methods was used for the investigation of the phase diagram. Phase equilibria, microstructure and chemical analysis of phases and overall compositions were performed by using scanning electron microscopy combined with energy dispersive X-ray spectroscopy (SEM–EDX), employing either a Zeiss Supra 55 VP instrument equipped with an energy dispersive detector for quantitative analysis or a similarly equipped SEM JEOL JSM-6460. Identification of phases present in the long-term annealed samples was achieved using X-ray powder diffraction. The Bruker D8 diffractometer equipped with a high-speed position sensitive (PSD) detector (Lynxeye) was used in the *θ*/2*θ* reflection setting. Rietveld refinements of selected diffraction patterns were performed with the Topas software [17]. Annealing temperature of the sample, overall composition, coexisting phases and phase compositions of the concrete phases in the equilibrium are listed in Table 2. Annealing temperature and number of the sample are listed in column 1, and the annealing time is shown in column 2. Column 3 shows the overall composition measured by SEM–EDX area scans. Coexisting phases stable in the samples are listed in column 4; columns 5-7 show the composition of equilibrium phases existing in the samples measured by SEM–EDX in same order as the phases are mentioned in column 4.

Phase transition temperatures were measured using a high-temperature DTA (NETZSCH Pegasus 404 C) with samples placed in closed and evacuated quartz glass DTA ampoules. Closed ampoules were used to limit uncontrolled Zn evaporation during the measurement and contamination of the inner parts of the instrument. The DTA was calibrated using a set of pure metal standards having well-defined melting temperatures (Sn, Al, Zn, Cu, Ag, Au). Calibration was carried out under the same conditions as the experimental measurements. Three heating and cooling runs were performed for each sample; the thermal effects during the first heating run were not taken into account. Thermal analysis results for four samples situated in the section *γ*′ - *γ* are listed in Table 3.

**Table 3:** Temperature of phase transitions measured by DTA

T [°C]_No.	Nominal comp. (at.%)			Thermal effects (heating) (°C)			
				Liquidus	Solidus	Transition *γ* ↔ *γ*′	other transitions
	Al	Cu	Zn				
400_38	18.6	53.7	27.7	940.7	903.8	569.1	
550_3	21.7	55.4	22.9	952.2	903.6	622.2	270.2
700_6	27.9	57.5	14.6	955.0	892.0	727.8	
700_4	32.8	56.8	10.4	*	*	826.9	641.9

*Has not been measured

## Results and discussion

**Figure 2:**
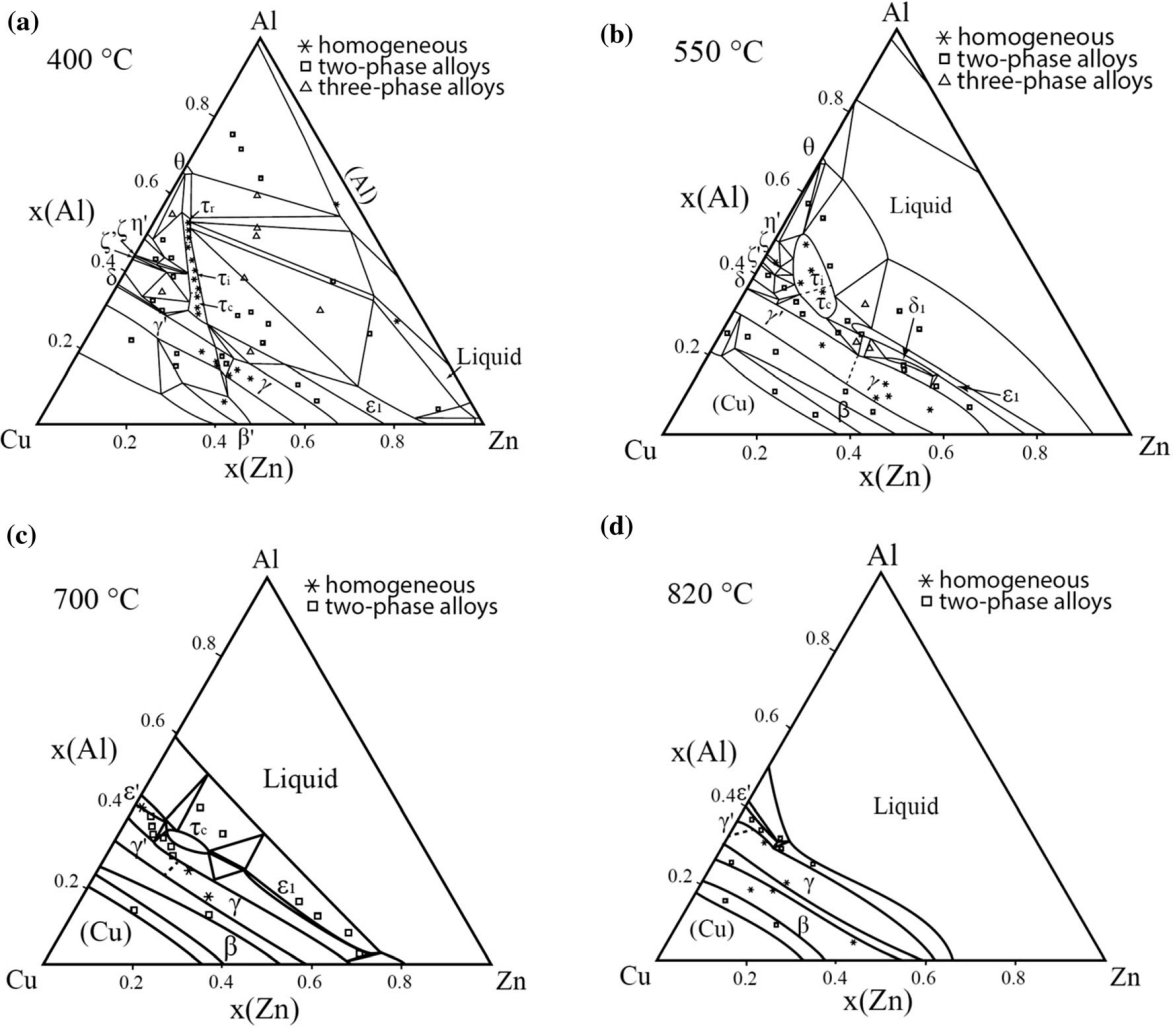
Isothermal sections of the Al–Cu–Zn experimental phase diagram at **a** 400 °C, **b** 550 °C, **c** 700 °C, **d** 820 °C. Overall compositions of selected samples are represented by several symbols. Stars represent the samples located in the single-phase field. Squares are the overall composition of the samples containing two phases in equilibrium. Compositions of each phase and relevant tie lines are not shown as the figure would be very difficult to read. Triangles represent the overall composition of the samples containing three phases in equilibrium. Phase compositions are defined by the corner of the tie triangle

By combining all experimental results listed in Tables 2 and 3, it was possible to draw complete isothermal sections of ternary phase diagram Al–Cu–Zn at 400 °C, 550 °C, 700 °C and 820 °C. These sections are presented in Fig. 2. The shape of the phase boundaries and phase fields not defined by our own samples was drawn based on information from binary subsystems, phase rules and data published by Ghosh et al. [1].

Our results generally agree well with the older phase diagrams published by Ghosh et al. [1], and the theoretical phase diagram published by Liang et al. [2], but contains some additional clarification and improvement of areas, which were not investigated in detail in the previous studies.

### Isothermal section at 400 °C

**Figure 3:**
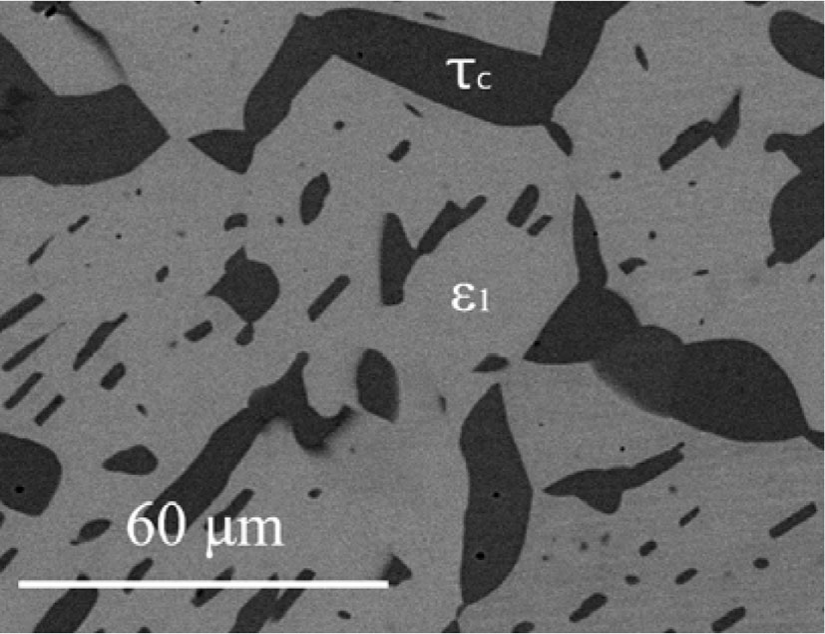
Microstructure of the sample 400_1 in BSE mode consist of *ε*_1_ and *τ*_c_ phases

**Figure 4:**
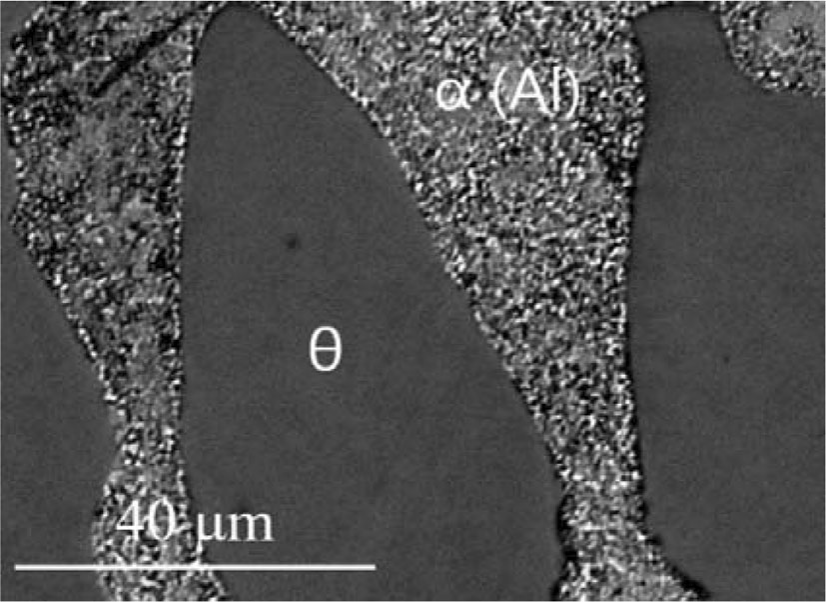
Microstructure of the sample 400_39 in BSE mode consist of *θ* and *α* (Al) phases

The isothermal section of phase diagram Al–Cu–Zn at 400 °C is shown in Fig. 2a. The ternary phase field *τ* exhibits a long line-shaped homogeneity range between the approximate compositions Al29Cu49Zn22 and Al48Cu42Zn10. In this area, we found at least three structural modifications (cubic form *τ*_c_, (presumably) incommensurate modification *τ*_i_ and rhombohedral *τ*_r_). The structural details are described in detail in the following paragraph. The Zn-poor region of the isothermal section shows equilibria of the *τ*-phase family with various binary Al–Cu compounds (phases *θ*, *η*′, ζ, *ζ*′, δ). The Al–Cu binary compounds in this central region show limited solubility of Zn. The solubility of Zn in the *θ*-phase is about 1 at.%. The phases *η*′, ζ, *ζ*′ show solubility of Zn about 5 at.% and δ phase about 10 at.%. *γ*′ phase has highest solubility of Zn up to 35 at.% of Zn. Based on binary phase diagram Cu–Zn, the ordered phase β’ is stable at 400 °C. Solubility of Al is up to 10%. The *γ* phase and *ε*_1_ phase have similar solubility of almost 20 at.% of Al. Figure 3 shows the microstructure of the two-phase sample *ε*_1_ + *τ*_c_. The liquid phase is stable around the binary eutectic point in Al–Zn and extends toward the more Al-rich compositions in the ternary with a solubility for Cu up to 5 at.%. The solubility of Cu in *α* (Al) solid solution increases with increasing Zn content reaches up to 5 at.%. The microstructure of the *α* (Al) phase in the two-phase field *α* (Al) + *θ* is not homogeneous (Fig. 4) due to the fact that the annealed samples go through the miscibility gap of *α* (Al) phase, and it decomposed to two *α* (Al) phases according the Al–Zn binary phase diagram (Fig. 1b).

#### The *τ*-phase

**Figure 5:**
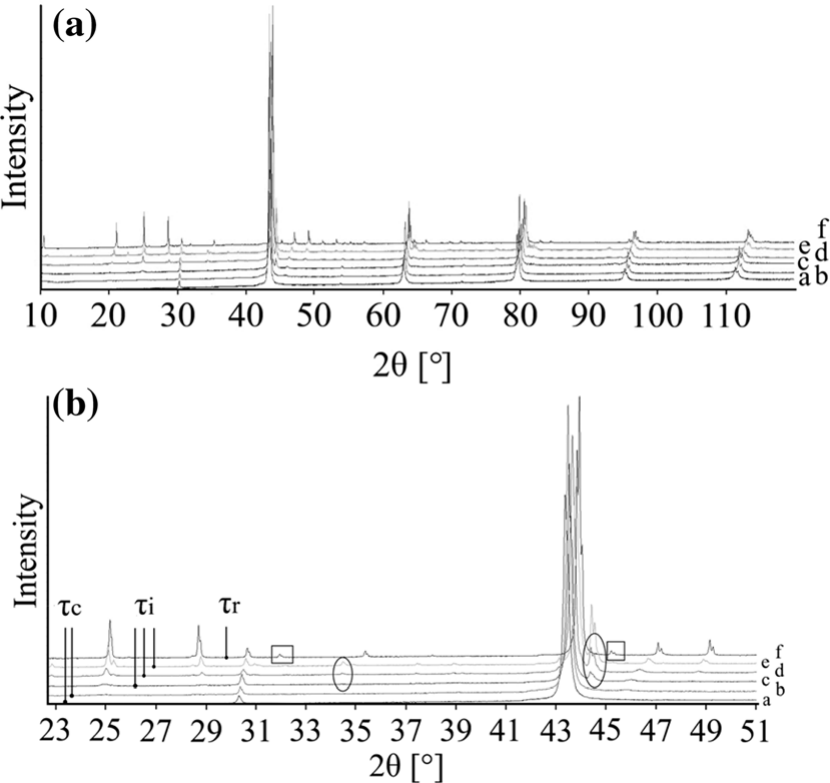
XRD patterns of the Al–Cu–Zn alloy containing *τ* phase. **a** whole measured range, **b** central part of the pattern. Specific peaks of *τ*_i_ phase are circled by ellipse. Specific peaks of *τ*_r_ phase are circled by square. Individual patterns were shifted on Y axe to better visualization. Overall compositions of the samples are following: *a:* 28.9 at.% Al–Cu-21.7 at.% Zn (*τ*_c_); *b:* 32.8 at.% Al–Cu-19.4 at.% Zn (*τ*_c_); *c:* 35.0 at.% Al–Cu-18.9 at.% Zn (*τ*_i_); *d:* 39.9 at.% Al–Cu-15.5 at.% Zn (*τ*_i_); *e* 45.4 at.% Al–Cu-11.0 at.% Zn (*τ*_i_); *f* 48.4 at.% Al–Cu-9.9 at.% Zn (*τ*_r_)

As mentioned above, the composition area of the *τ*-phase is crystallographically complex. Originally, two different modifications of the phase were reported in the literature [18]: *τ* with CsCl-type structure and *τ*′ with a closely related rhombohedral structure space group *R*-3 *m,* Pearson symbol *hR*30. In the latter structure, six atomic sites are present, of which three being occupied by the Cs atoms and three by the Cl in specific sublattices of B2. Distortions from the ideal cubic coordination CN = 14 (8 + 6) are only small. Our detailed evaluation of the powder patterns of single-phase samples in the 400 °C isothermal section yielded the following results: The cubic B2 structure was found in samples situated at the Cu- and Zn-rich end of the homogeneity range (*τ*_c_), while the rhombohedral structure was found at the opposite end of the homogeneity range (*τ*_r_). The intermediate composition range, here designated as (*τ*_i_), could not be refined properly, although all samples were single phase according to SEM results. In this area, additional superstructure reflexes were observed which could not be indexed with any reasonable set of cell parameters. Position and intensity of superstructure reflexes varied continuously with the composition, but the number of observed superstructure reflexes increased with decreasing Cu content. This behavior leads to the conclusion that this intermediate area probably contains an incommensurately modulated crystal structure related to both, the B2 structure and its rhombohedral counterpart. It is not clear if the different phase regions *τ*_c_, *τ*_i_ and *τ*_r_ are separated by two-phase fields, however, in spite of the large number of samples investigated in this area, it was not possible to identify any composition gap in the phases field. Consequently, the different areas are only separated by dashed lines in Fig. 2a. XRD patterns of some selected samples containing the family of *τ* phases are shown in comparison in Fig. 5.

Samples containing *τ*_c_ and *τ*_r_ were further investigated by Rietveld refinement in order to reveal the site occupations. For *τ*_c_, a site occupation model allowing vacancies on the Cu site and Al/Zn substitution on the Al site yielded excellent agreement with the sample composition obtained by EDX measurements (Table 4). This defect mechanism is well in line with other nonstoichiometric B2 intermetallics like e.g., NiAl showing vacancies on the transition metal site [19].

**Table 4:** Comparison of phase compositions for *τ*_c_ and *τ*_r_ determined by Rietveld refinements and EDX

Sample	Phase	Method	at.% Al	at.% Cu	at.% Zn
400_13	*τ*_c_	EDX	32.7	47.8	19.5
		Rietveld	29.9	48.5	21.6
400_46	*τ*_c_	EDX	30.4	49.0	20.6
		Rietveld	31.6	49.0	19.4
400_86	*τ*_c_	EDX	28.9	49.5	21.6
		Rietveld	28.8	49.5	21.7
400_11	*τ*_r_	EDX	48.4	41.7	9.9
		Rietveld	54.2	40.0	5.8
400_34	*τ*_r_	EDX	50.9	40.3	8.8
		Rietveld	54.9	39.8	5.3

The rhombohedral structure is much more complicated, so site occupation refinements were more demanding. Table 5 summarizes the refinement results for sample 400_34 in the single-phase region *τ*_r_. Free refinement of Cu occupation factors yielded the two fully occupied positions Cu1 and Cu2, while the occupation of Cu3 is only 0.15. The position Al2 is fully occupied by Al, while the two positions Al1 and Al3 show partial substitution with Zn. The resulting calculated overall composition based on Rietveld refinement is in reasonable agreement with results from EDX concentration measurements; a second sample in the *τ*_r_ region yielded comparable results (Table 4).

**Table 5:** Crystallographic parameters for the phase *τ*_r_ in sample 400_34 obtained by Rietveld refinement

Space group *R*-3 *m*, Pearson symbol *hR*30						
*a* = 4.126569(18) Å, *c* = 25.19380(15) Å						
Site	Wyckoff Pos.	*x*	*y*	*z*	Occupation	B_eq_
Cu1	3*a*	1/3	2/3	1/8	Cu: 1.00(14)	0.878(30)
Cu2	6*c*	2/3	1/3	0.030544(50)	Cu: 1.00(14)	0.878(30)
Cu3	6*c*	0	0	0.10673(35)	Cu: 0.153(20)	0.878(30)
Al1	6*c*	1/3	2/3	0.06919(12)	Al: 0.90(11) Zn: 0.10(11)	0.678(94)
Al2	6*c*	2/3	1/3	0.132822(92)	Al: 1.000(94) Zn: 0.000(94)	0.678(94)
Al3	3*a*	0	0	0	Al: 0.76(13) Zn: 0.24(13)	0.678(94)

### Isothermal section at 550 °C

**Figure 6:**
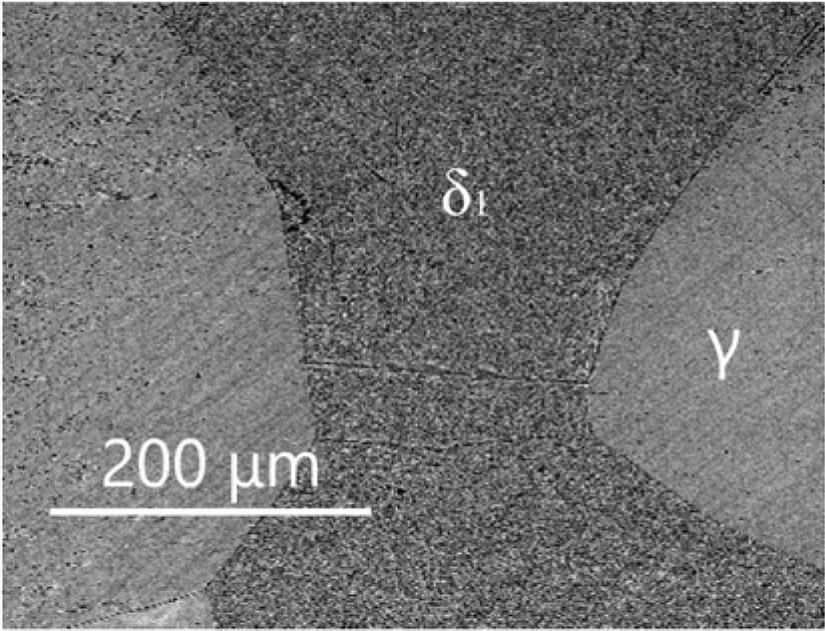
Microstructure of the sample 550_35 in BSE mode consists of *γ* and δ_1_ phases

**Figure 7:**
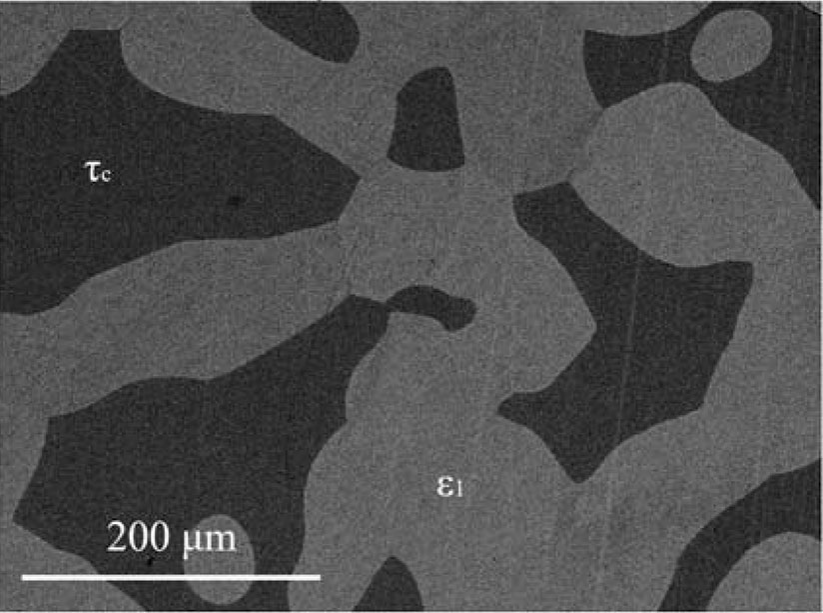
Microstructure of the sample 550_18 in BSE mode consists of *ε*_1_ and *τ*_c_ phases

The isothermal section of phase diagram Al–Cu–Zn at temperature 550 °C is shown in Fig. 2b. At this temperature, only *τ*_C_ and *τ*_i_ were identified—the rhombohedral structural modification was not observed. The pseudo-ternary phase δ_1_ is found at 550 °C in equilibrium with *γ* (Fig. 6). Thus, the binary δ_1_ phase in the Cu–Zn system is stabilized toward lower temperatures by the addition of Al. Binary Al–Cu compounds (phases *θ*, *η*′, ζ, *ζ*′, δ) from the central part of the binary phase diagram are in equilibrium with the *τ*-phase family with only limited solubility of Zn. This part of the phase diagram is quite complex and phase relations drawn in Fig. 2b are based on diagrams published by Ghosh et al. [1]. The *θ*-phase shows solubility of Zn about 1 at.%. The phases *η*′, *ζ*, *ζ*′, δ show solubility of Zn about 5 at.%. The *γ*′ phase is stable up to 30 at.% of Zn. At this concentration, a second-order transition between the *γ* and *γ*′ phases exists. Details of evaluation are discussed together with the vertical section in "The γ/γ′ phase field" section. The disordered phase β is stable at 550 °C, and it shows high solubility of Al up to 20 at.%. *ε*_1_ phase has very high solubility at almost 30 at.% of Al, close to the ternary *τ*_c_. Figure 7 shows the microstructure of a sample in the two-phase field *τ*_c_ + *ε*_1_. The liquid phase is stable from Al–Zn binary phase diagram up to 30 at.% of Cu, and it is connected to the eutectic point of the Al–Cu system.

### Isothermal section at 700 °C

**Figure 8:**
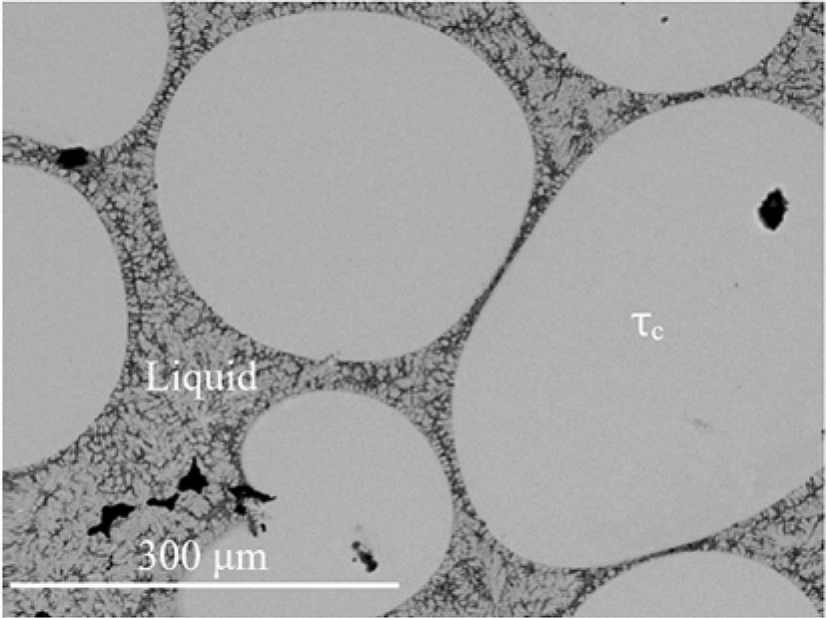
Microstructure of the sample 700_14 in BSE mode consists of liquid and *τ*_c_ phases

**Figure 9:**
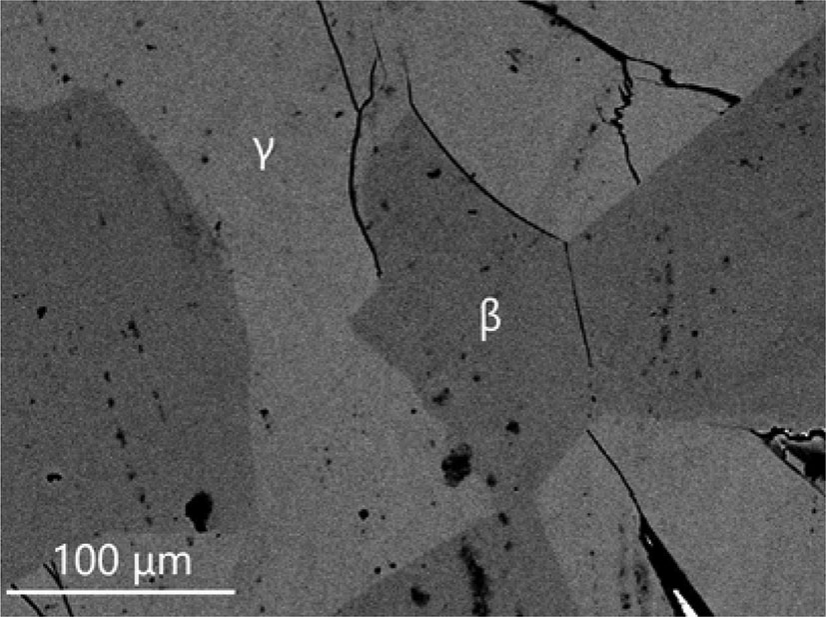
Microstructure of the sample 700_12 in BSE mode consists of β and *γ* phases

The isothermal section of phase diagram Al–Cu–Zn at 700 °C is presented in Fig. 2c. At this temperature, the cubic modification of *τ* phase is the only remaining modification. Figure 8 shows a micrograph of the two-phase field *τ*_c_ + L. The liquid phase is stable from Cu-80Zn to Al-40Cu and covers the whole Cu poor concentration range. Pseudo-ternary *ε*_1_ was found stable between *γ* and the liquid phase. Phase *ε*′ has a solubility up to 5 at.% of Zn close to the phase *τ*_c_. The second-order transition between *γ* and *γ*′ is found around 20 at.% Zn. The β phase exhibits a complete solubility from Al–Cu to Cu–Zn. Figure 9 shows β phase in equilibrium with *γ* phase.

### Isothermal section at 820 °C

**Figure 10:**
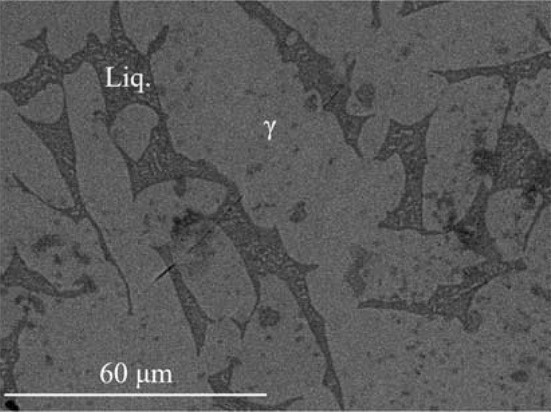
Microstructure of the sample 820_11 in BSE mode consists of liquid and *γ* phases

**Figure 11:**
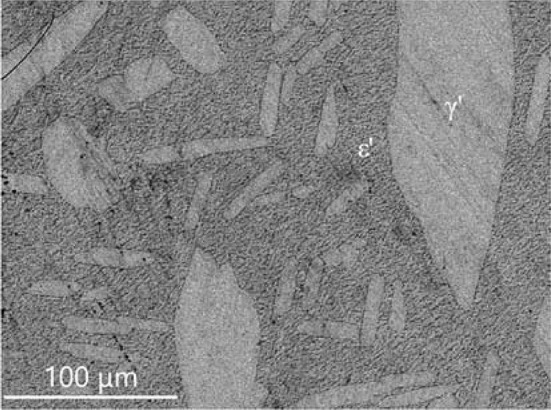
Microstructure of the sample 820_1 in BSE mode consists of *ε*′ and *γ*′ phases

The isothermal section of phase diagram Al–Cu–Zn at 820 °C is presented in Fig. 2d. The temperature of 820 °C was chosen because at this temperature the *γ* phase is stable in Al–Cu as well as in the Cu–Zn phase diagram. Figure 10 shows the microstructure of a *γ* + *L* equilibrium. Full mutual solubility between the two binary *γ* phases has been expected and was confirmed. On Al-rich part of *γ*-family phase field was found *γ*′ phase with very low solubility, this is in agreement with binary Al–Cu phase diagram. We did not find any ternary or pseudo-ternary phase stable at 820 °C. The *ε*′ phase shows solubility of Zn up to 15 at.%, and it is shown in equilibrium with *γ*′ in Fig. 11.

### The *γ*/*γ*′ phase field

**Figure 12:**
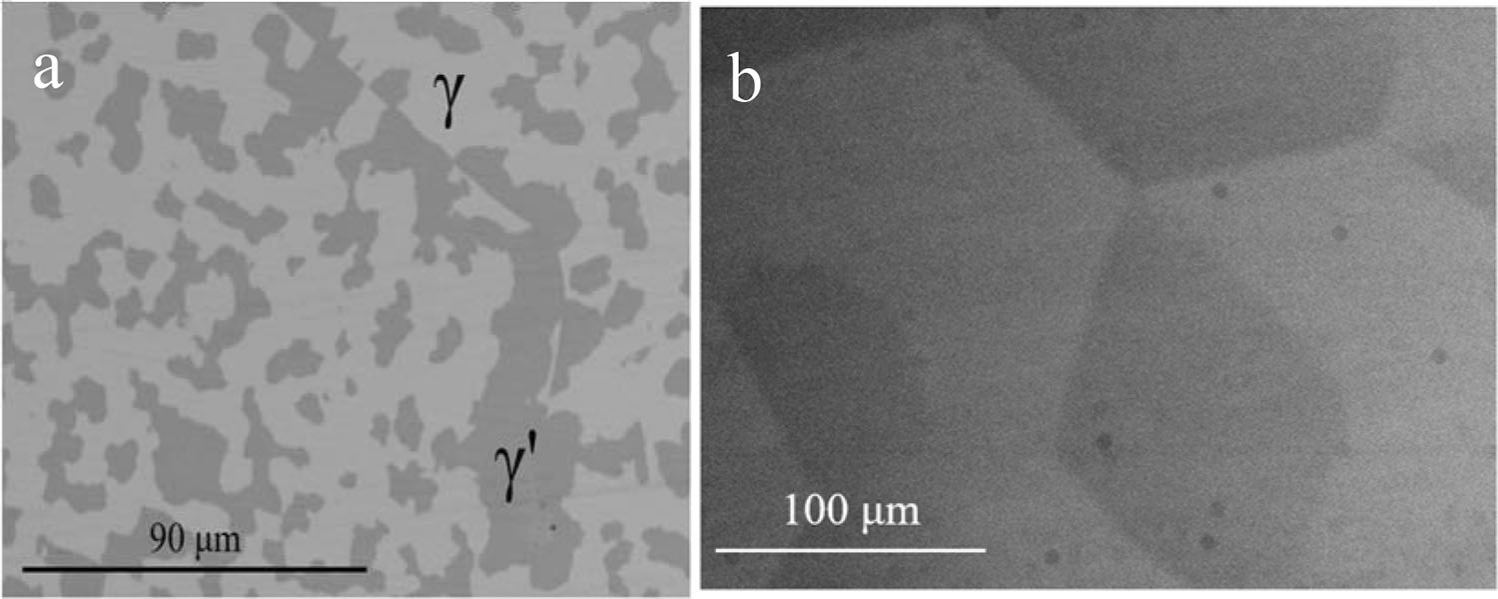
Micrographs in BSE mode of **a** sample 350_1 with overall composition 14.9 at.% Al-48.2 at.% Cu–Zn that had been annealed at 350 °C containing well-crystallized phases *γ* + *γ*′ in equilibriums, **b** sample 450_1 (15.9 at.% Al-50.9 at.% Cu–Zn) annealed at 450 °C without two-phase structure

**Figure 13:**
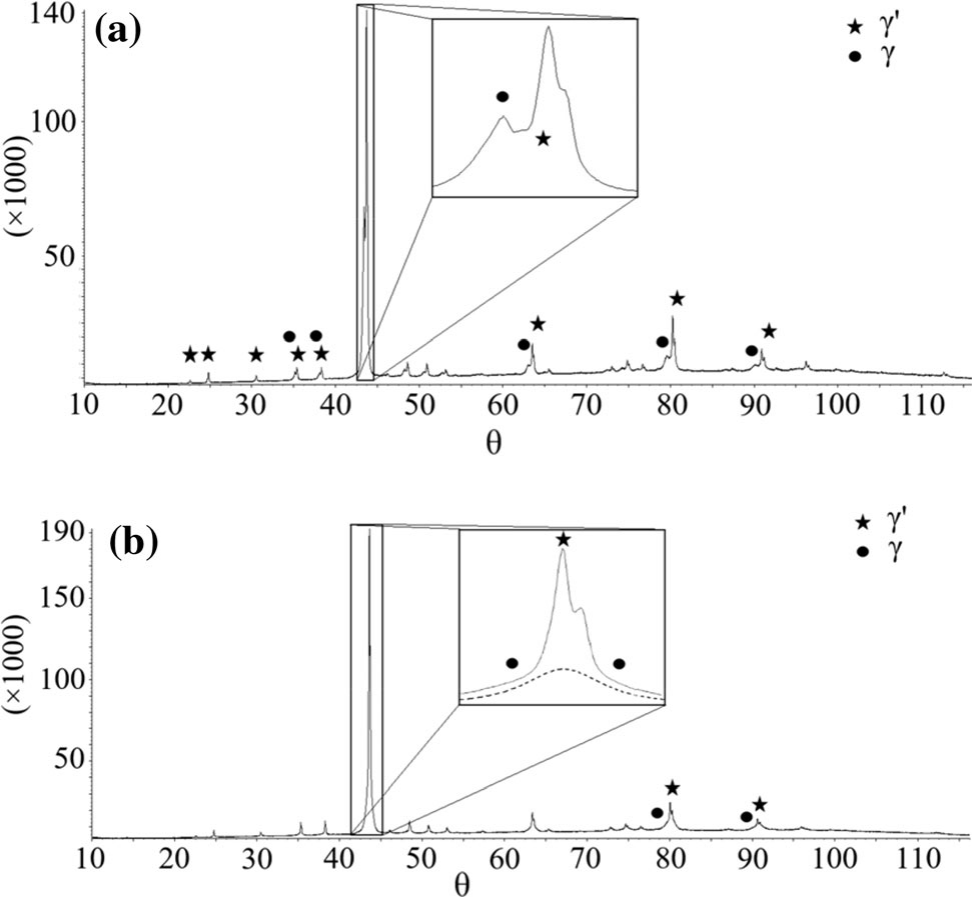
XRD pattern of the alloy Al–Cu–Zn containing. **a** well-crystallized phases *γ* and *γ*′ in the sample 14.4 at.% Al-48.8 at.% Cu–Zn annealed at 300 °C (300_1) and **b** poorly crystallized phase *γ* in the sample 15.9 at.% Al-50.9 at.% Cu–Zn annealed at 450 (450_1)

One of the major goals of the current study was to define the phase equilibrium relations between *γ* (*cI*52) and *γ*′ (*cP*52). As mentioned in the previous sections, a small two-phase field *γ* + *γ*′ was found at 400 °C, while two different structures were identified in different samples at 550 °C and 700 °C (see detailed description below), but no two-phase field was found. In order to define the *γ* + *γ*′ two-phase field better, a set of additional samples with composition close to the 15 at.% Al-49 at.% Cu–Zn was prepared and long-term annealed at different temperatures at and below 400 °C. Samples annealed at the temperatures of 300 °C, 325 °C, 350 °C, 375 °C and 400 °C show well-crystallized and well-separated two phases *γ* + *γ*′ in equilibrium (see microstructure on Fig. 12a and XRD pattern Fig. 13a). In contrast to this, samples 15.9 at.% Al-50.9 at.% Cu–Zn annealed at 450 °C, 14.4 at.% Al-48.9 at.% Cu–Zn annealed at 500 °C and 12.4Al-46.3Cu–Zn annealed at 550 °C contained large grains in different orientation (see microstructure on Fig. 12b), but were found to be homogeneous in terms of composition. Their XRD patterns (see e.g., Fig. 13b) showed well-defined diffraction lines of the primitive structure of *γ*′ in combination with a significant broadening of the basis of all lines fulfilling the reflection conditions for the base-centered structure. Consequently, it was possible to refine the pattern well by assuming an overlay of well-crystallized primitive *γ*′ (refined crystalline domain size 212 nm) and base-centered *γ* of poor crystalline quality (refined crystalline domain size 24 nm). This leads to the conclusion that these samples were actually single phase at the temperature of annealing and partially transformed during quenching.

**Figure 14:**
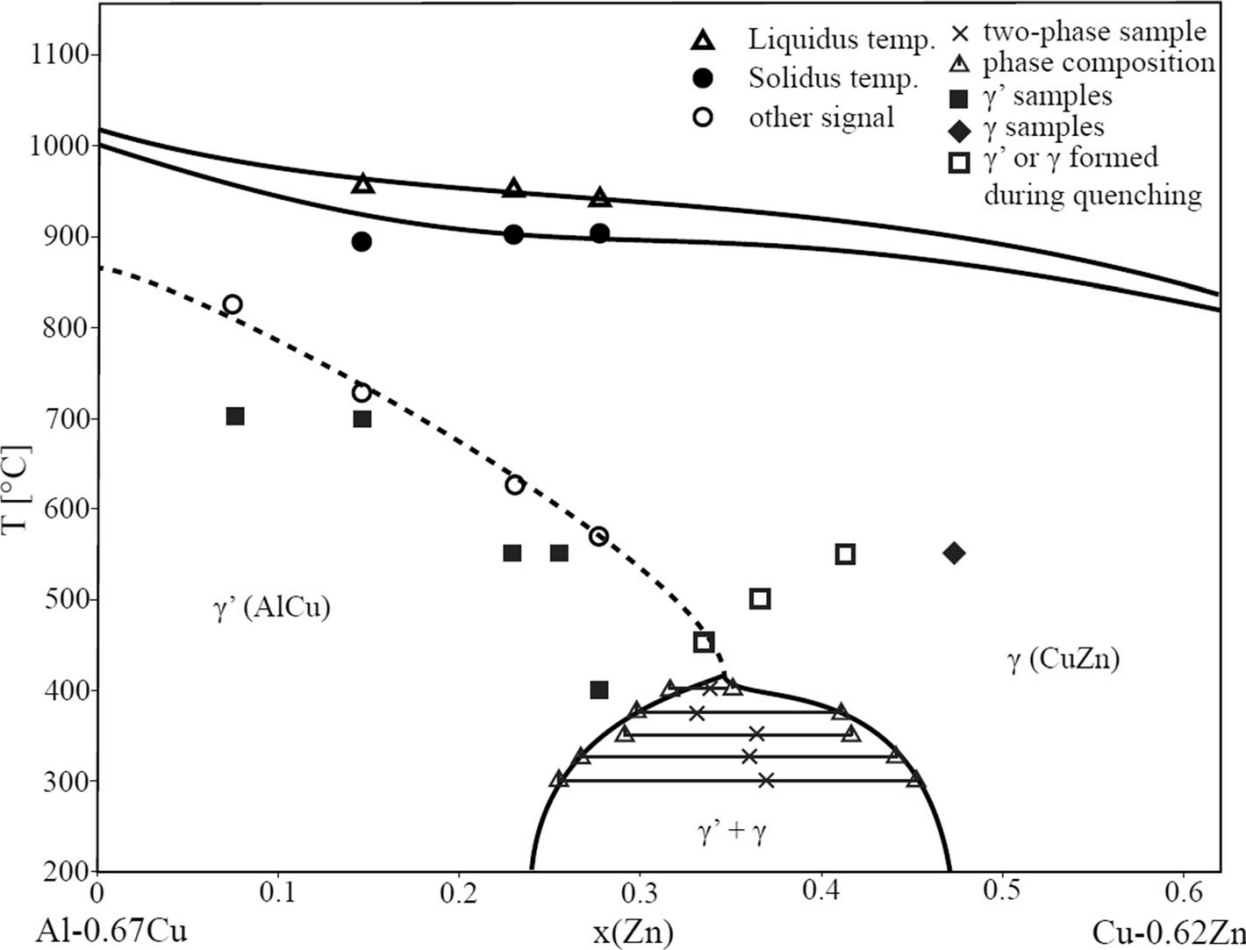
Vertical section of the ternary phase diagram Al–Cu–Zn between Al–0.67Cu and Cu–0.62Zn

This conclusion was actually confirmed by plotting the results from all samples into a vertical section as shown in Fig. 14. The *γ*′ + *γ* two-phase samples are represented by the tie lines terminated by triangles with overall composition marked by cross symbol. The two-phase gap is getting smaller with increasing temperature and appears to close above 400 °C. Samples showing the characteristically broadened XRD pattern (open squares) are situated in the single-phase region but entered the two-phase field during quenching. Single-phase samples showing *γ* (diamond) or *γ*′ (full squares) without specific broadening did not show the characteristic line broadening.

In order to confirm the proposed second-order transition line above approximately 440 °C shown as dashed line in Fig. 14, additional DTA experiments were performed on four samples situated in the respective composition area.

**Figure 15:**
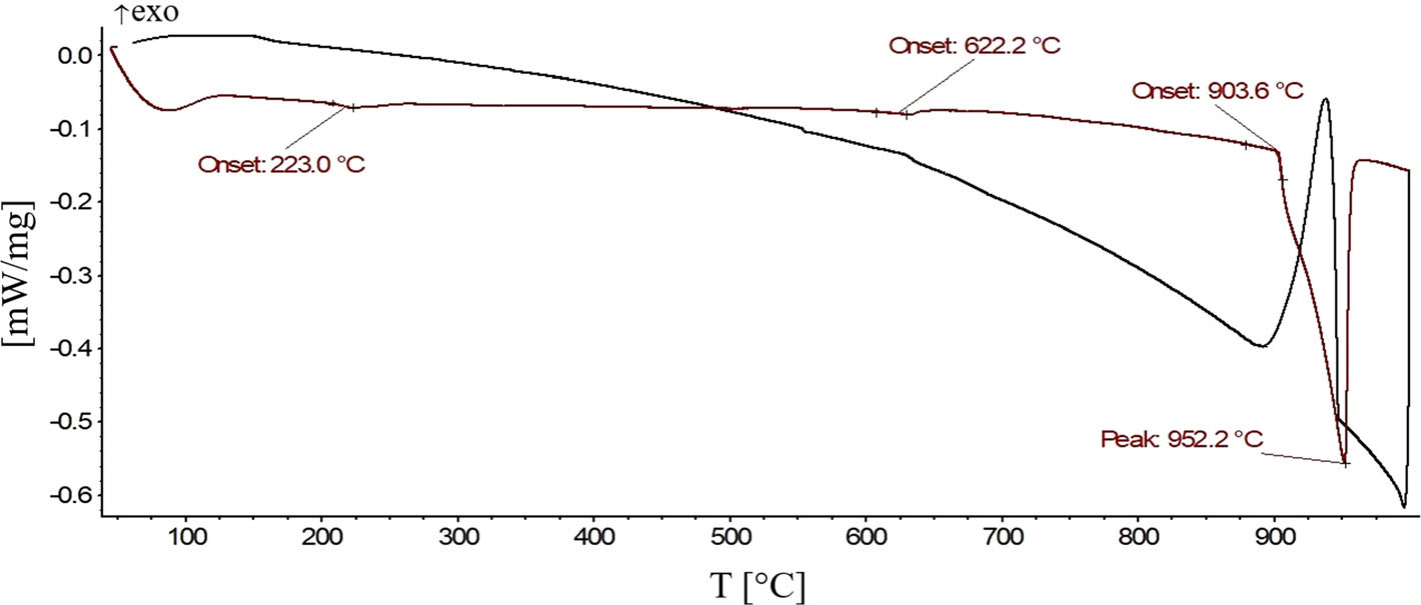
The third DTA heating and cooling curves of the 21.7 at.% Al-55.4 at.% Cu-Zn sample. Signals correspond to the temperature of the second-order reaction *γ* ↔ *γ*′ (632.4 °C) and to the liquidus temperature (952.2 °C). Measurement conditions: sample was placed in sealed evacuated quartz glass ampoule, inert atmosphere 5 N Ar 50 ml min^−1^, heating rate 5 °C min^−1^

Measurements were performed under a permanent Ar flow of 50 ml min^−1^ and with heating and cooling rates of 5 °C min^−1^. Three runs were performed for each sample; the thermal effects during the first heating run were not taken into account. The temperature of the thermal effects used was thus the average value of the thermal effects of the second and third heating curves only. Small differences between the first and subsequent heating and cooling curves are caused by changes in the shape of the sample following initial melting. Temperature of phase transition in solid phase was evaluated as an onset of peak, liquidus was evaluated as a minimum of peak. The results of the DTA third heating and cooling curves of the sample with composition 21.7 at.% Al-55.4 at.% Cu–Zn is presented in Fig. 15. Signals correspond to the temperature of the second-order reaction *γ* ↔ *γ*′ at 622.2 °C (open circles on Fig. 14), to solidus temperature at 903.8 °C (filled circles on Fig. 14) and to the liquidus temperature at 952.2 °C (triangles on Fig. 14). Composition of the samples was checked after DTA measurement to ascertain that the sample did not react with the SiO_2_ material of the ampoules. Thermal analysis results are listed in Table 3, and the transition temperatures for the *γ* ↔ *γ*′ transition are shown as open circles in Fig. 14.

The interpretation of the dashed line separating the *γ* and *γ*′ phase fields as second-order reaction *γ* ↔ *γ*′ is well in line with the observed DTA effects. It also agrees with experimental results from binary phase diagram Al–Cu, where authors [3, 20] proposed a second-order transition between the primitive and base-centered structure there.

## Conclusions

Although the literature related to the Al–Cu–Zn phase diagram is numerous, some of the complex phase equilibria were not well solved. The current study was designed to contribute to a better understanding of those parts of the phase diagram that needed improvement and refinement. The experimental studies were carried out at temperatures 400 °C, 550 °C, 700 °C and 820 °C, and some additional measurements were done also at additional temperatures in the *γ* + *γ*′ phase region. This was achieved by a combination of standard methods: overall and phase compositions of samples were measured using SEM–EDX, the temperatures of phase transitions by DTA measurements in evacuated quartz glass DTA ampoules. The crystal structures were identified by XRD.

The following main results were obtained in present study:

- Mutual relationships of *γ* + *γ*′ phases were studied in whole concentration and temperature range. Independent two-phase field of *γ* + *γ*′ was observed up to 400 °C. At higher temperature, the phase transition *γ* ↔ *γ*′ is proposed to be second order.
- An isopleth between the binary phases *γ* and *γ*′ was constructed
- For the ternary phase, at 400 °C we found strongly temperature-dependent one phase fields containing phases with cubic CsCl-type structure (*τ*_c_) and a related rhombohedral structure type (*τ*_r_), respectively, and an intermediate composition range with apparently incommensurate modulation (*τ*_i_). The rhombohedral structure type (*τ*_r_) was not found at 550 °C and above. At 700 °C we only found the cubic structure modification (*τ*_r_)
- Isothermal sections at 400 °C, 550 °C, 700 °C and 820 °C were constructed
